# Children’s Learning of Non-adjacent Dependencies Using a Web-Based Computer Game Setting

**DOI:** 10.3389/fpsyg.2021.734877

**Published:** 2021-11-03

**Authors:** Mireia Marimon, Andrea Hofmann, João Veríssimo, Claudia Männel, Angela D. Friederici, Barbara Höhle, Isabell Wartenburger

**Affiliations:** ^1^Cognitive Sciences, Department of Linguistics, University of Potsdam, Potsdam, Germany; ^2^Early Childhood Education Research, University of Applied Sciences, Potsdam, Germany; ^3^School of Arts and Humanities, University of Lisbon, Lisbon, Portugal; ^4^Department of Neuropsychology, Max Planck Institute for Human Cognitive and Brain Sciences, Leipzig, Germany; ^5^Department of Audiology and Phoniatrics, Charité – Universitätsmedizin Berlin, Berlin, Germany

**Keywords:** non-adjacent dependencies, rule learning, web-based, implicit learning, serial reaction time (SRT) task, SRT

## Abstract

Infants show impressive speech decoding abilities and detect acoustic regularities that highlight the syntactic relations of a language, often coded *via* non-adjacent dependencies (NADs, e.g., *is singing*). It has been claimed that infants learn NADs implicitly and associatively through passive listening and that there is a shift from effortless associative learning to a more controlled learning of NADs after the age of 2 years, potentially driven by the maturation of the prefrontal cortex. To investigate if older children are able to learn NADs, Lammertink et al. (2019) recently developed a word-monitoring serial reaction time (SRT) task and could show that 6–11-year-old children learned the NADs, as their reaction times (RTs) increased then they were presented with violated NADs. In the current study we adapted their experimental paradigm and tested NAD learning in a younger group of 52 children between the age of 4–8 years in a remote, web-based, game-like setting (*whack-a-mole*). Children were exposed to Italian phrases containing NADs and had to monitor the occurrence of a target syllable, which was the second element of the NAD. After exposure, children did a “Stem Completion” task in which they were presented with the first element of the NAD and had to choose the second element of the NAD to complete the stimuli. Our findings show that, despite large variability in the data, children aged 4–8 years are sensitive to NADs; they show the expected differences in r RTs in the SRT task and could transfer the NAD-rule in the Stem Completion task. We discuss these results with respect to the development of NAD dependency learning in childhood and the practical impact and limitations of collecting these data in a web-based setting.

## Introduction

To acquire their native language, infants not only have to learn the words but also the rule-based relations between the individual words, which make up the syntax of that language. Some of these grammatical rules, known as non-adjacent dependencies (NADs), consist of statistically reliable relationships between two speech elements separated by intervening elements. An example from English is the morphological relation between an auxiliary *is* and a verb suffix *-ing* in *My brother is dancing*. The ability to extract and track NADs from speech is crucial for language acquisition (Kidd and Arciuli, 2016; Lany and Shoaib, 2020). Infants have been shown to be able to learn NADs from passive listening (e.g., Gómez, 2002; Gómez and Maye, 2005; Friederici et al., 2011; Marchetto and Bonatti, 2013; for a review see Wilson et al., 2018). However, this learning seems to be hindered under certain conditions, for example, if the variability of the intervening element is low (Gómez, 2002), if the NADs are embedded in complex passages (Santelmann and Jusczyk, 1998), or if the stream does not contain any mark for segmentation (Marchetto and Bonatti, 2013). Although NAD learning might continue to be difficult for children in their second year of life it becomes more and more sophisticated over development (e.g., across phonological word boundaries, generalization to new contexts, more complex patterns, etc.). For instance, infants from 17 months of age show NAD learning even when the discrimination required was extremely subtle (e.g., *pel kicey rud* vs. *pel kicey jic*) (Gómez and Maye, 2005, for English-learning infants) and even when the auxiliary and verb suffix crossed a phonological phrase boundary (van Heugten and Shi, 2010, for French-learning infants). Later, at 19 months of age, infants can recognize NADs over two intervening syllables (Höhle et al., 2006, for German-learning infants). Adults have been shown to be successful NAD learners when tested under passive listening conditions with behavioral methods (Uddén et al., 2012; Frost and Monaghan, 2016; Wang et al., 2019). However, evidence from electrophysiological and neuroimaging studies using identical materials and task settings have shown differences between adults’ and infants’ NAD learning from passive listening. These studies are outlined in the next paragraph.

Friederici et al. (2011) showed that already 4-month-old German-learning infants can track NADs in an unfamiliar natural language, namely Italian. The authors measured event-related potentials (ERPs) while infants heard Italian sentences consisting of a noun phrase followed by an NAD (e.g., *Il fratello sta cantando*, the brother is singing). The stimuli alternated familiarization (learning) and subsequent test phases which contained the familiarized NADs and violations of the NADs (e.g., ^∗^*Il fratello sta cantare*, ^∗^the brother is sing; ^∗^means agrammatical). Results showed a broad positive-going ERP component in response to the NAD violations indicating that infants could discriminate between familiarized and violated NADs after a short period of passive listening, and thus were sensitive to NADs. This P600-like positivity was similar to the response of adult native Italian speakers, but differed from German speaking adults, who showed an N400 effect that was taken as evidence for lexical, rather than syntactic processing (Mueller et al., 2009). Only after a prolonged time of exposure (Citron et al., 2011) or explicit instructions (Mueller et al., 2012) German speakers’ brain responses showed a similar pattern as the ones of the native speakers.

For NAD learning in early childhood, Mueller et al. (2019) reported that 2-year-olds, but not 4-year-olds showed ERP markers of rule learning from passive listening. van der Kant et al. (2020) further narrowed down the period of this developmental change and showed that NAD learning undergoes a qualitative change between 2 and 3 years of age. Their results indicated learning of NADs *via* passive listening for children at the age of 2 years, but not at the age of 3 years. In line with these findings, it has been proposed that the ability to learn implicitly (i.e., without instruction and/or feedback) from passive listening declines from early infancy to later childhood (Skeide and Friederici, 2016). The question arises as to whether the capacity for a more associative bottom-up learning from passive listening ends abruptly around the age of 3 years or whether it might gradually be replaced by a more top-down, controlled learning mechanism. Paul et al. (2020) investigated this transition of NAD learning and collected ERP data from children between 1 and 3 years of age. Using the same experimental paradigm as in the above cited studies (Mueller et al., 2009; Citron et al., 2011; Friederici et al., 2011) they observed that the amplitude of the ERP effect of NAD learning decreased linearly with age suggesting a gradual decrease of NAD learning from passive listening. Importantly, Paul et al. (2020) argued on the one hand for a developmental shift, presumably influenced by maturation of the prefrontal cortex (PFC) and other neuronal circuits (Skeide and Friederici, 2016), but on the other hand also proposed that children’s knowledge and entrenchment of their native language has an influence on the changes in their learning outcomes. According to Skeide and Friederici (2016), when maturation has reached a certain degree, top-down control increasingly takes effect, which in turn inhibits associative bottom-up learning mechanisms, also limiting the ability to learn NADs under passive listening. In line with this idea, Friederici et al. (2013) demonstrated that in a passive listening experiment, in which adults’ left prefrontal region was downregulated with a cathodal transcranial direct current stimulation, they showed a late positivity for violated NADs similar to infants, indicating associative learning. In the control sham-condition, adults showed the lexical N400-like component as in Mueller et al. (2009). The developmental shift from more associative to more controlled learning mechanisms thus seems to be related to the development of the PFC functions.

So far, there has been little evidence for this developmental decline in NAD learning from behavioral paradigms. One of the possible hurdles is that behavioral data collection in children is limited: grammaticality judgments (e.g., two-alternative-forced-choice task, 2AFC), reaction time (RT) and reflection-based measures (Isbilen et al., 2017), typically used with adult participants, are challenging for children (Lammertink et al., 2019). Lammertink et al. (2019) developed a promising methodological setting to examine children’s NAD learning behavior by adapting a serial reaction time (SRT) task combined with a word-monitoring task for children aged 5;9 to 8;6 years (see also López-Barroso et al., 2016). In this children-friendly game setting (in the lab), participants were introduced to two little monkeys on a computer screen and were asked to help the monkeys gather bananas, while they were exposed to an artificial language string containing items with and without NADs. Children were then asked to press a button as fast as possible if they heard a specific target syllable (Version 1: target “lut” in “tep X lut;” Version 2: target “mip” in “sot X mip;” X stands for 72 variable elements between the NADs) and another non-target button if the target syllable was not presented. During the initial “learning blocks,” children reacted faster over time in response to the target- and non-target syllables. But crucially, they slowed down in the so-called “reversal block” (in the present study we name it “disruption block”), in which the second element of the NAD (“lut” in Version 1 and “mip” in Version 2) was not preceded by the first element of the learned NAD (“tep” or “sot,” respectively), but by a novel syllable. After that, children were presented with the correct NADs (“recovery block”) and again, were faster in their responses. These results showed that children were sensitive to the NADs, because the first element predicted the second element, resulting in this specific RT pattern. In addition, children completed a grammaticality judgment task (2AFC), a more explicit task that tests for abstraction and transfer of the NAD to a new setting. However, in this task children performed only at chance level. The authors argued that, although 2AFC measures are widely used to test NAD learning, the required degree of metalinguistic or explicit knowledge may have influenced the judgments, possibly invalidating their use with children. This is corroborated by Bialystok (1986), who indicates that metalinguistic skills are acquired and mastered not until the age of 7 years.

In the present study, we aim to replicate and extend Lammertink et al.’s (2019) study by examining the ability to learn NADs in younger children (4–8-year-olds) in a web-based active SRT learning task with natural language stimuli (adapted from Friederici et al., 2011). As in Lammertink et al. (2019), we employed an active task, as we asked participants to interact and actively press buttons in response to the presented stimuli. In addition, we adapted the 2AFC task of Lammertink et al. (2019) to test the abstraction and transfer of the internal rule to a new setting. Thus, our study aims to address (1) whether implicit learning of NADs in 4–8-year-old children can be captured by means of RTs in an active web-based task, (2) whether NAD learning can additionally be measured by means of a Stem Completion (SC) task, and (3) whether children’s NAD learning (in either task) is modulated by age. Importantly, our work differs from Lammertink et al. (2019) in the following aspects:

### Age Range

Existing literature on NAD learning has either mainly explored early infancy up to the age of 4 years (e.g., Gómez, 2002; Mueller et al., 2019; Paul et al., 2020; van der Kant et al., 2020), older children (e.g., 6–8-year-olds; Lammertink et al., 2019), or adults (e.g., Frost and Monaghan, 2016; Arnon, 2019) with data on children between 4 and 6 years still missing. However, there is evidence that a developmental shift happens gradually between 2 and 4 years of age (Mueller et al., 2018; Paul et al., 2020; van der Kant et al., 2020). To systematically investigate the question of how NAD learning trajectories unfold and what influences the magnitude of NAD learning beyond the age of 3 years, we collected behavioral data in children from 4 to 8 years of age. So far, to the best of our knowledge, no empirical data of SRT learning measures for NAD learning or the combination of RTs and response accuracy measures exists for children across the whole the age range of 4–8 years (besides the partial overlap with Lammertink et al., 2019).

### Stem Completion Task

Lammertink et al. (2019) included a 2AFC task in which children heard pairs of utterances and had to decide which of the two utterances was most familiar to the artificial language heard in the previous word-monitoring task (e.g., “tep X lut” or “tep X mip”). However, children did not exceed chance level in this task. In our study we included an SC task instead. Children heard only the first part of the NAD without the final element (e.g., “sta cant-”) and were asked to decide which ending would fit best (i.e., target syllable, “ando” or non-target syllable “are”) by clicking on the respective button on the keyboard. Thus, our task is still a decision task, but it includes two main deviations: firstly, children are not presented with the alternative options and then forced to choose between these two, but rather must decide on the best possible completion of the stimulus from two possible “hidden” options (“are” or “ando”) without hearing the “complete” stimulus. Secondly, with this approach we did not have to create a new task environment with completely new instructions, but the children had to continue behaving in a similar manner as in the SRT task, that is, monitoring the target syllable. However, we consider this task more explicit than the SRT task, because participants need to access the previously learned underlying NAD rule.

## Natural Stimuli

We used natural language stimuli (adapted from Friederici et al., 2011) instead of artificial syllable strings or phrases (e.g., “tep X lut,”; Lammertink et al., 2019). Experiments using artificial languages have received criticism in recent years in terms of ecological validity (Yang, 2004; for a review see Erickson and Thiessen, 2015). Compared to natural language, artificial languages are relatively simple in their acoustic properties and contain less variability that defines rhythm and stress characteristics.

### Web-Based Study

Finally, due to the worldwide pandemic situation (COVID-19), our study was fully run at home on an Internet browser instead of in the laboratory. Whereas web-based data collection in adults is extensively and successfully used and several well-established RT effects have been replicated in web-based research (Crump et al., 2013; Simcox and Fiez, 2014), there are only a few recent web-based studies with children and infants (Scott et al., 2017; Nussenbaum et al., 2020; Rhodes et al., 2020; Bambha and Casasola, 2021; Vales et al., 2021). Recent studies collecting RTs with adults and children have shown little to no difference between laboratory-based and web-based samples (de Leeuw and Motz, 2016; Hilbig, 2016; Bridges et al., 2020; Nussenbaum et al., 2020; Morini and Blair, 2021; Silver et al., 2021; Vales et al., 2021) as well as no big differences between browsers (e.g., Chrome and Internet Explorer) or experiment builders (e.g., Pavlovia and Gorilla) (Kochari, 2019; Anwyl-Irvine et al., 2020; Sauter et al., 2020). In addition, it is difficult to clearly state whether potential differences may be any greater than the difference between two laboratory-based collected samples (Nussenbaum et al., 2020). Furthermore, our study was unmoderated, which means that there was no interaction with the experimenter and children needed minimal assistance from parents. The feasibility of collecting web-based data with children and young infants in unmoderated studies has recently been demonstrated (Scott et al., 2017; for a discussion of the advantages and challenges, see Rhodes et al., 2020; Bambha and Casasola, 2021). Web-based data collection allows to collect larger sample sizes which leads to increased statistical power (Brand and Bradley, 2012; Nussenbaum et al., 2020).

Based on the previous literature, our hypotheses are that all children should be able to learn NADs within the word-monitoring SRT task (learning from passive listening). Specifically, we expected a training effect, a disruption effect and a recovery effect. The **training effect** would be confirmed if children’s RTs decreased through the first exposure learning blocks, in which only correct (i.e., to-be-learned) NADs are presented. A **disruption effect** would be confirmed if, as in Lammertink et al. (2019), RTs increased during the disruption block in which children are presented with violated NADs (according to what they have learned in the previous learning blocks). Finally, the expected **recovery effect** would be confirmed if RTs decreased again after shifting back to the correct, to-be-learned NADs (recovery block; same stimuli as in the initial learning blocks). In the subsequent SC task, children had to apply the NAD rule learned during the SRT task. Thus, in this task we test whether the implicitly learned rule of the SRT task can be transferred by the participants to a more explicit knowledge. In addition, a positive correlation between both tasks would indicate that better NAD-learners can better extract and transfer the underlying NAD rule to a different task demand. Finally, we expected that age would modulate the three effects in the SRT task and the accuracy in the SC task. Since the ability to learn from passive listening might decline due to the increased top-down control of the more mature PFC (Friederici et al., 2013; Skeide and Friederici, 2016), older children may show less sensitivity to the NADs in our stimuli than younger children.

## Materials and Methods

### Participants

Overall, 91 monolingual German-speaking children fully completed the first part of the experiment (SRT task). Twenty-three additional children started the study and quit before the first task was finished and therefore their data were not included in the analysis. From the sample of 91 children, 37 children responded randomly on the two buttons [i.e., showed at-chance performance in the word-monitoring task of the SRT part (see section “Data Preprocessing”)]. In line with the exclusion criteria of Lammertink et al. (2019), data of these children were excluded from the analysis. Two further participants were excluded because they did the task twice. Two additional datasets were excluded only from the SC task analysis due to incomplete data. In the final sample, a total of 52 children were included (27 girls and 25 boys; age range: 3;7–8;04 years; mean = 6.21 years, SD = 1.12 years)^1^ (see Table 1). Participants were recruited through the BabyLab database and the internet portal *Kinder Schaffen Wissen*^2^ and received a compensation of five Euros if requested. Before starting the study, parents reported no speech or hearing disorders for their children and no daily exposure to a Romance language (French, Italian, Spanish, Romanian, or Portuguese) and gave their consent. Ethical approval was obtained from the Ethics Committee of the University of Potsdam (EA 43/2018).

**TABLE 1:** Summary of participants’ characteristics that were included in the data analysis.

Total	Age in years^a^	Handedness (total)	Other languages (total)	Time to finish study in minutes^a^
27	Female			
	6.05 (1.03, range = 4.01–7.37)	Right (23) Left (2) NA (2)	English (2) Finnish (1) Polish (1)	19.94 (6.88, range = 15.83–30.94)
25	Male			
	6.34 (1.19, range = 3.70–8.04)	Right (23) Both (2)	Russian (2)	23.10 (7.40, range = 16.83–46.99)

*^*a*^Mean (SD, range).*

### Stimuli

All stimuli (adapted from Friederici et al., 2011) were recorded by a native Italian speaker and consisted of short grammatical and ungrammatical Italian utterances of the form AXB. Here, the A-element represents the first element of the NAD and refers to two different auxiliaries depending on the experimental version: an Italian verb auxiliary (*sta*) or an Italian modal verb (*può*). The middle element X was a variable Italian verb stem. Each verb stem was morphologically marked and contained one of two Italian suffixes (B-element -*are* or -*ando*, depending on the version). Grammatical and ungrammatical NAD stimuli were generated by combining each auxiliary with each suffix and cross-splicing (see Friederici et al., 2011, for a detailed description; see stimuli used here in Table 2). Thus, the NAD stimuli contained a monosyllabic A-element (*sta* or *può*) followed by one monosyllabic verb stem (one out of 32 different X-elements), followed by a bisyllabic B-element (*are* or *ando*) (e.g., *sta cantando* and *può cantare*).

**TABLE 2:** Stimuli and stimulus specifications.

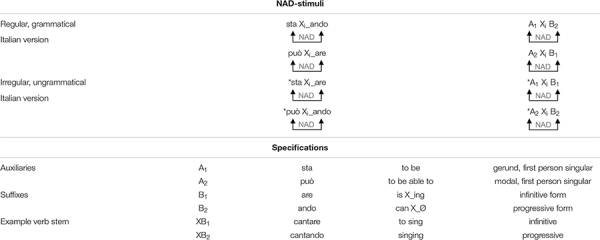

*means agrammatical.

**FIGURE 1:**
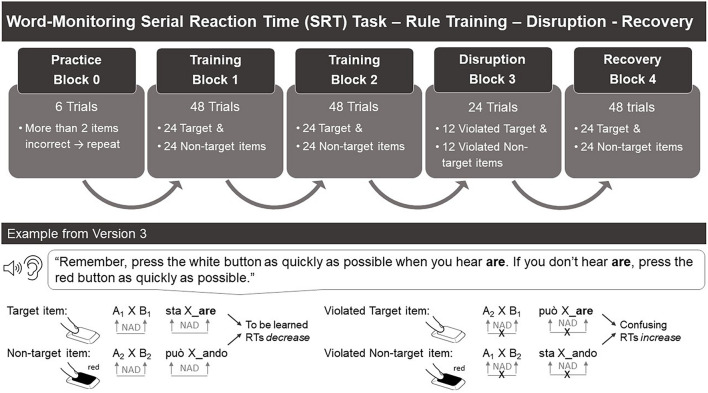
Block types within word-monitoring SRT task, trial and item distribution, and an example draft of the procedure.

The B-element (*are* or *ando*) was the target syllable for the word-monitoring task. The target syllables and the corresponding NADs were counterbalanced across participants. A total of 4 experimental versions were created and counterbalanced across participants to control for any intrinsic biases and saliency toward the native Italian grammatical dependencies (see Table 3 for all combinations). Thus, half of the participants learned the NADs *sta-X-ando* and *può-X-are;* and for half of them *ando* was the target syllable, for the other half *are* was the target syllable. The other half of the participants learned the NADs *può-X-ando* and *sta-X-are;* and for half of them *ando* was the target, for the other half *are* was the target. For the sake of simplicity, we explain the tasks for only one of these four different lists below (Version 3, see Figure 1).

**TABLE 3:** Trial types and their use within blocks and each version of the word-monitoring SRT task.

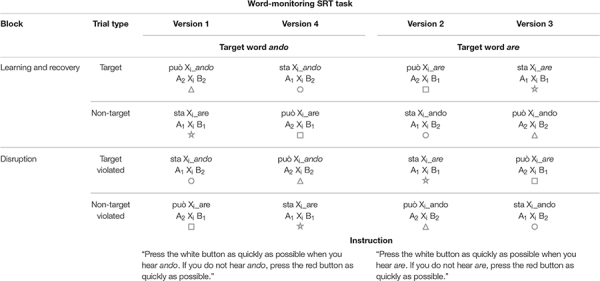

Out of the 32 different Italian verb stems (X-elements), 24 were randomly selected and present in both NADs (e.g., *sta-X-ando* and *può-X-are*) and appeared in a different order in each block. Therefore, in the SRT task, children were presented with these verb stems twice in the learning blocks and the recovery block and once in the disruption block (hence called familiar verb stems). The SC task contained eight trials with familiar verb stems and eight trials with the remaining novel verb stems that participants did not hear during the SRT task.

Each stimulus contained a silent pause of 20 ms at the beginning and a pause between auxiliary and verb stem (pause in *può* stimuli: mean = 259 ms, min = 148 ms, max = 350 ms; pause in *sta* stimuli: mean = 261 ms, min = 142 ms, max = 361 ms), but no pause between verb stem and suffix. Table 4 contains all verbs with the respective suffix used in the study. The average trial length for *sta X_ando* trials was 1550 ms (SD = 66 ms), *for sta X_are* trials it was 1450 ms (SD = 70 ms), for *può X_ando* trials it was 1410 ms (SD = 61 ms), and for *può X_are* trials it was 1320 ms (SD = 55 ms). There was no significant difference in the B-element onset (*ando* and *are*) across the stimuli (*p* = 0.90). All auditory instructions for the game were recorded by a female native speaker of German.

**TABLE 4:** Overview of the 32 verb stems with the respective suffix combinations, used within stimuli.

Infinitive	Gerund
amare, andare	amando, andando
bagnare, ballare, bussare	bagnando, ballando, bussando
cantare, cercare, chiamare, cullare	cantando, cercando, chiamando, cullando
danzare	danzando
entrare	entrando
filmare, fischiare	filmando, fischiando
gelare, gettare, giocare, girare, graffiare, gridare	gelando, gettando, giocando, girando, graffiando, gridando
lodare	lodando
mangiare, mostrare	mangiando, mostrando
ornare	ornando
pagare, pappare, passare, pensare, picchiare	pagando, pappando, passando, pensando, picchiando
stirare, suonare	stirando, suonando
tirare	tirando
volare	volando

*Each verb stem was combined once with sta and once with può, depending on the experimental version.*

#### Serial Reaction Time Task

The word-monitoring SRT task included a practice block, an exposure phase consisting of two learning blocks followed by a disruption block and a recovery block (Figure 1). The practice block consisted of six trials and was fully repeated if children responded incorrectly in more than two trials. Each learning block consisted of 48 NAD trials (24 target and 24 non-target trials). The disruption block after the learning blocks consisted of 24 trials (12 violated target and 12 violated non-target trials) and was followed by a recovery block of 48 trials (24 target and 24 non-target trials). Every 24 trials children received feedback on the number of stars (correct responses) collected so far.

The stimuli presented in the different blocks were counterbalanced across four different trial types: *target trial, non-target trial, violated target trial*, and *violated non-target trial*. A target trial contained the target syllable that children were asked to monitor during the experiment by pressing the button when hearing it (e.g., *are*). The non-target trial was therefore determined by the absence of the target syllable: if the target trial contained the target syllable *are*, the non-target trial contained the non-target syllable *ando*. For example, the target syllable in Version 3 was *are*. Hence, a target trial in Version 3 was *sta*-X-*are* and a non-target trial was *può*-X-*ando*. These trials were presented during both the learning blocks and the recovery block. In the disruption block, the trials presented contained only “disrupted” NADs (violated target trials and violated non-target trials). In these types of trials, the dependency between the first and the second NAD element was violated. For example, a violated target trial in Version 3 was *può*-X-*are* and a violated non-target trial was *sta*-X-*ando.* Thus, the violated target trials still contained the target syllable that participants had to monitor (i.e., *are* in our example from Version 3). Therefore, during the SRT task, the children had always to monitor the same target syllable, which was assigned to the same button throughout the experiment.

#### Stem Completion Task

Stimuli used in this task consisted only of the first element of the NAD and the verb stem (AX-element, e.g., *può cant-*) and did not contain the second element of the NAD (B-element, *ando* or *are*), which was cut at zero-crossing from each utterance using the software *Praat* (Boersma and Weenink, 2018). All edited auditory stimuli for the SC task were checked by 17 different adult raters unaware of the experimental procedure to evaluate whether the missing suffix could somehow be derived from the stimuli (e.g., because of subtle coarticulation differences). The raters listened to each stimulus individually and selected through a questionnaire whether -*are* or -*ando* was a better fit at the end. All rater assignments were at chance level and response biases due to inherent stimulus characteristics could therefore be ruled out (*N* = 32 possible correct assignments, *p* = 0.5 probability of success, range of chance level between 12 and 20 correct assignments, actual ratings: between 14 and 19 correct assignments). In the SC task, the participants had to select the correct completion of the NAD by pressing on the target button or non-target button, respectively. For example, in Version 3, in which children were asked to monitor the target syllable *are*, a target trial consisted of *sta cant-.* The children would need to press the target button, as *are* would be the correct answer according to the learned NAD. A non-target trial consisted of *può cant-* and the children would need to press the non-target button, as *ando* would be the correct answer according to the previously learned NAD. The SC task consisted of 16 trials (8 target and 8 non-target) and included feedback (stars) only at the end of the task.

### Procedure

The experiment was accessed *via* a link on the BabyLab page of the University of Potsdam and was programmed and deployed on the web-based *LabVanced* software^3^ (Finger et al., 2017). Before starting the experiment, parents were asked to prepare a white and a red button by placing a white sticker on the P key and a red one on the Q key (under the assumption that parents with children at home have paper, colored pencils, and sticky tape at hand). At the beginning of the experiment, parents were asked to fill in a short questionnaire about their child (month and year of birth, sex, handedness, input in other languages, and speech and hearing impairment). After that, parents were requested to test and adjust the volume on the speakers or headphones. Parents were asked not to get involved with the experiment, but to stay close by. After the parental questionnaire, the experiment started and children were introduced to two different cartoon moles, Mali and his brother Max, who invited the child to play a catching game with them (*whack-a-mole*; Nissen and Bullemer, 1987; Qian et al., 2016). For this, children were instructed to listen very carefully because they would listen to phrases from a secret language (the NADs) which sometimes included a specific target syllable and sometimes not. Children were instructed to press the white target button (right hand) as soon as they heard the specific target syllable and the red non-target button (left hand) otherwise. Children were also told that they had to answer questions about the secret language at the end. The instruction was then briefly repeated so that the target syllable (*ando* or *are*) and the corresponding required responses were remembered before a practice block of the SRT task started. RTs were recorded as the dependent variable. At the beginning of each trial, the two moles appeared next to each other and the audio containing a single NAD phrase (e.g., *sta cantando*) was directly played. The child could press the button anytime from stimulus onset. As soon as the child pressed one of the two buttons, the feedback appeared, which depended on the accuracy of the child’s response. If the child responded correctly (i.e., pressed the target button if the target syllable was present or pressed the non-target button if the target syllable was not present), the mole on the respective side was caught with a net. As an additional reward, a star appeared and a sound was played (positive feedback). If the child responded incorrectly (i.e., pressed the target button if the target syllable was not present or pressed the non-target button if the target syllable was present), only an empty net appeared in the middle between the two moles and no sound was played. After the child responded, the next trial started with the two moles and a new trial containing an NAD phrase. Pressing the button was self-paced, there was no trial timeout and therefore no null responses were recorded. After the SRT task ended, the instructions for the SC task were explained to the participants. Children were told that they would hear phrases from the secret language again but without an ending. They were asked to choose the best ending (either *are* or *ando*) with the same button press procedure as before and were told that they would receive a star reward at the very end. They were asked to guess if they were unsure. Response accuracy was collected as the dependent variable. A trial in the SC task started the same way as in the SRT task. However, the feedback differed: a red or a white circle appeared on the mole according to the participants’ response and independently of the accuracy of the answer.

All children had to perform both tasks immediately after each other, preferably without a pause. At the very end, parents were asked to indicate whether the children used headphones during the whole experiment. On average, children took 21.6 min to complete the experiment (SD = 7.32, range = 15.83–46.99 min). Each participant used their own laptop or desktop^4^ and participants were asked to wear headphones if available. The caregiver of 12 children reported that their child wore headphones throughout the entire experiment and three did so only for the SRT task; the others reported that they used loudspeakers. All collected metrics were provided by *LabVanced* as a downloadable csv file. The experiment, in digital JSON format, along with all scripts used, can be found at our Open Science Framework project page.

## Data Analysis

### Data Preprocessing

Following Lammertink et al. (2019), we included only data of those participants in the analysis who were able to follow the instruction of the word-monitoring task and did not respond randomly using the two buttons in the two learning blocks and the recovery block in the SRT task. Hence, an above-chance performance in the word-monitoring task was considered an indication of adequate task compliance. Monitoring the target syllable (i.e., whether the syllable appeared in a sentence) was therefore considered a “secondary” task, which was a relatively easy and cognitively low demanding compared to the main task that consisted in implicitly learning the internal structure of the NADs (see section “Participants”). In the final sample only correct target word monitoring responses were analyzed (78.29% of total number of trials). In addition, three criteria were applied to determine outliers and exclude individual RT data points. First, RTs lower or equal to 200 ms were removed (1.9% of total number of trials), because RTs up to 200 ms from stimulus onset may be too fast to reflect the processes of interest, as they correspond to the approximate duration to plan and execute an adequate motoric response (Dahan et al., 2019). Secondly, since there was no timeout for trials, RTs above 7000 ms were considered long RT outliers based on visual inspection of the data (e.g., Ratcliff, 1993; Baayen and Milin, 2010), as they are more likely not revealing any information about the underlying linguistic processing, and therefore they were removed (2.3% of total number of measures). This specific cut-off was chosen *post hoc* after observing the large variability of the data. Finally, RTs that were 2.5 SD above or below the mean RT for the corresponding target type (target, non-target, violated target, and violated non-target) of the same participant in the same block were removed as well (2.3% of total number of trials). A total percentage of 5.2% of individual RTs were excluded from further analysis based on the described criteria. The final dataset contained 6335 observations, all for correct responses only, distributed over four blocks (first learning block: 1676 out of 1840 observations; second learning block: 1826 out of 1953 observations; disruption block: 917 out of 981 observations; and recovery block: 1916 out of 2065 observations). In the SC task, responses were coded as correct or incorrect (accuracy). A correct response (coded as 1) was possible in two ways: (a) if children pressed the target button in a target trial, deciding that the target syllable would be the best fit for completion of the NAD or (b) if children pressed the non-target button in a non-target trial, deciding that the non-target syllable would be the best fit for completion of the NAD. In all other cases the response was incorrect (coded as 0).

### Statistical Analysis

We based our analyses on Lammertink et al. (2019), who kindly provided their scripts on OSF.^5^ Both RTs and accuracy data were analyzed in R (R Core Team, 2017) using (generalized) linear mixed-effects models (*lme4* package; Bates et al., 2015). Confidence intervals were calculated using the profile method (provided within *lme4* package), odds ratios and probabilities were calculated following the script of Lammertink et al. (2019) and *p*-values were obtained by loading the *lmerTest* package in R before fitting the model (Kuznetsova et al., 2017). All corresponding figures were generated using the *ggplot2* package (Wickham, 2009). The raw RTs of the final dataset were log-transformed. This was determined by an assessment of the best Box–Cox power transform, a procedure that allows selecting the appropriate data transformation that normalizes the residuals of the statistical models (Box and Cox, 1964; Kliegl et al., 2009; Kowarik, 2019).

#### Serial Reaction Time Task

We employed a linear mixed-effects model in which log RT was the dependent outcome variable in the model. In the model, *Block* was entered as a fixed effect with four levels: first learning block, second learning block, disruption block, and recovery block. To obtain information about the three effects of interest we used successive difference contrasts that allowed us to directly test the difference between condition means of neighboring block levels. With the generated contrasts, the difference between two successive blocks is tested while condition means for the other block levels is ignored (Schad et al., 2020). The corresponding comparison between mean (log-scaled) RTs made it possible to confirm the presence of the following effects: first, a **training effect**, which contained the difference between the first learning block and second learning block (coded as first learning block = −0.75, second learning block = +0.25, and remaining *Block* levels = +0.25). Second, a **disruption effect**, which contained the difference between the second learning block and the disruption block (coded as second learning block = −0.5, disruption block = +0.5, first learning block = −0.5, and recovery block = +0.5). Finally, a **recovery effect** was determined through the difference between the disruption block and the recovery block (coded as disruption block = −0.25, recovery block = +0.75, and remaining blocks = −0.25). *Targetness* (target and non-target) was entered as a fixed effect and coded as a sum-to-zero contrast (difference of the level means between target, coded +0.5, and non-target, coded −0.5, see Schad et al., 2020). *Age* (in years and months) was centered and included as a continuous variable. The model contained random intercepts by-subject (*Subject*) and by-item (*Item*) and random slopes by-subject for the main effects of *Targetness* and *Block*, as well as for the interaction between *Targetness* and *Block*, and a random slope by-item for *Age*. Since we modeled fixed effects for all predictors with sum-to-zero contrasts, it allowed us to estimate the respective coefficients as overall effects across the levels of all other predictors, defining the intercept term as the grand mean across all predictor levels. The model structure was selected prior to data collection and was based on Lammertink et al.’s (2019) approach with deviations in the contrast coding and without *Version* as a predictor. To determine a sensible random-effects structure we used a backward-selection heuristic (Matuschek et al., 2017) based on the AIC criterion (Akaike, 1998) to arrive at a model that included the random effects’ structure justified by the data without losing goodness-of fit and without losing power to detect fixed effects or substantially increase Type I error rates. Note that the resulting model is highly complex and may therefore lack power to detect any of the interaction effects, especially three-way interactions.

#### Stem Completion Task

We employed a generalized linear mixed-effects model (mixed-effects logistic regression model) in which accuracy was the dependent outcome variable. We fitted a model to estimate whether children scored better in items with a familiar verb stem compared to items with a novel verb stem (*Familiarity*), and whether children scored better on target trials compared to non-target trials (*Targetness*). The model included two binary predictors, each interacting with *Age* as a covariate. *Familiarity* and *Targetness* were included as fixed factors and both were coded with sum-to-zero contrasts (verb stem: familiar +0.5, novel −0.5; item: target +0.5, non-target −0.5). *Age* (in years and months) was centered and included as a continuous variable. The model contained only by-subject (*Subject*) random intercepts and slopes for the main effect of *Targetness*. The parsimonious random effects’ structure was derived by means of a backward-selection heuristic (Matuschek et al., 2017), as in the SRT task model. Finally, we computed a Pearson’s correlation coefficient to determine the relationship between the SRT and the SC task. The outcome of the SRT task was calculated by subtracting a child’s average log-transformed RT in the disruption block from his/her log-transformed RT average in the second training block and recovery block (i.e., disruption peak). The SC score was the number of correct responses from each child.

## Results

### Serial Reaction Time Task

**FIGURE 2:**
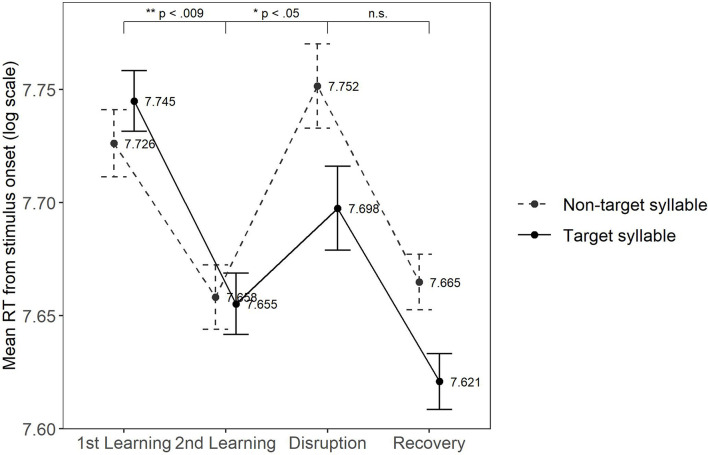
Mean response times from stimulus onset (RTs in log-scale) and error bars with SEs for the target (solid) and non-target (dashed) syllable across the four blocks of exposure. The numerical values for the means are annotated. ^∗^Indicates statistical significance, as indicated by the *p*-value.

We tested whether children’s RTs showed the three effects of interest (training effect, disruption effect, and recovery effect). Figure 2 shows the mean RTs across the blocks according to *Targetness* (target and non-target). The complete output of the model is provided in Table 5. The results indicated a statistically significant effect for responses collapsed across both target types for two effects of interest: the training effect and the disruption effect. The **training effect** in the model output shows that children were 156.22 ms (back-transformed from model estimates) faster in the second learning block compared to the first one [*t* = −2.74; *p* = 0.006; 95% CI (−0.13, −0.02)]. The **disruption effect** was indicated by a 103.56 ms increase in RTs in the disruption block compared to the second learning block [*t* = +2.04; *p* = 0.04; 95% CI (0.00, 0.09)]. The **recovery effect** was not statistically significant: the mean RTs on log scale in the recovery block were 83.81 ms shorter than in the disruption block [*t* = −1.75; *p* = 0.08; 95% CI (−0.09, 0.00)]. In addition, the output of the model showed a main effect of Targetness: children were faster responding to target items than to non-target items [−44.37 ms, *t* = −2.31; *p* = 0.02; 95% CI (−0.04, 0.00)]. Finally, we found a main effect of Age [*t* = −2.66; *p* = 0.01; 95% CI (−0.16, −0.02)], suggesting that overall older children responded faster than younger children (−186.91 ms on average), and an interaction between Age and Targetness [*t* = −2.02; *p* = 0.04; 95% CI (−0.03, 0.00)]. Finally, we found a main effect of *Age* [*t* = −2.66; *p* = 0.01; 95% CI (−0.44, −0.07)], suggesting that overall older children responded faster than younger children (−186.91 ms on average), and an interaction between *Age* and *Targetness* [*t* = −2.02; *p* = 0.04; 95% CI (−0.09, −0.00)]. We divided the group into three subgroups according to age and we observed three different types of behavior: younger children seemed to be slower for target items compared to non-target items, the middle age subgroup seemed to show no difference in their responses when presented with either target type, and older children seemed to be faster for targets compared to non-target items. However, *Age* did not significantly modulate the effects of interest (i.e., training effect, disruption effect, and recovery effect). No other significant effects were found (see Table 5).

**TABLE 5:** Summary of the RT model (6335 observations; *N* = 52).

	RT model – log RT				

Predictors	Estimates*	SE*	CI*	*t*-Statistic	*p*-Value
(Intercept)	7.66	0.04	(7.59 to 7.74)	195.18	**<0.001**
Training effect	−0.08	0.03	(−0.13 to −0.02)	−2.74	**0.006**
Disruption effect	0.05	0.02	(0.00 to 0.09)	2.04	**0.041**
Recovery effect	−0.04	0.02	(−0.09 to 0.00)	−1.75	0.080
Targetness	−0.02	0.01	(−0.04 to −0.00)	−2.31	**0.021**
Age (centered)	−0.09	0.03	(−0.16 to −0.02)	−2.66	**0.008**
Training effect* Targetness	−0.01	0.02	(−0.06 to 0.04)	−0.44	0.662
Disruption effect* Targetness	−0.03	0.04	(−0.11 to 0.06)	−0.61	0.541
Recovery effect* Targetness	−0.01	0.03	(−0.08 to 0.06)	−0.30	0.765
Training effect* Age (centered)	0.04	0.02	(−0.01 to 0.09)	1.59	0.111
Disruption effect* Age (centered)	0.03	0.02	(−0.01 to 0.07)	1.46	0.143
Recovery effect* Age (centered)	−0.02	0.02	(−0.06 to 0.02)	−1.05	0.293
Targetness* Age (centered)	−0.02	0.01	(−0.03 to −0.00)	−2.02	**0.043**
Training effect* Targetness* Age (centered)	−0.00	0.02	(−0.05 to 0.04)	−0.24	0.813
Disruption effect* Targetness* Age (centered)	0.03	0.04	(−0.04 to 0.11)	0.87	0.384
Recovery effect* Targetness* Age (centered)	0.01	0.03	(−0.05 to 0.07)	0.24	0.808

**Random effects**					

σ^2^	0.08	τ_1 Item.c_age_	0.00	ρ_01 Item_	−0.91
τ_0 Item_	0.00	τ_1 Subj.trainingEffect_	0.04	ρ_01 Subj.trainingEffect_	0.35
τ_0 Subj_	0.08	τ_1 Subj.disruptionEffect_	0.02	ρ_01 Subj.disruptionEffect_	0.05
N _Item_	96	τ_1 Subj.krecoveryEffect_	0.02	ρ_01 Subj.krecoveryEffect_	−0.41
N _Subj_	52	τ_1 Subj.Targetness_	0.00	ρ_01 Subj.Targetness_	−0.55
Observations:	6335	τ_1 Subj.trainingEffect:Targetness_	0.01	ρ_01 Subj.trainingEffect:Targetn_	0.26
Marginal *R*^2^	0.152	τ_1 Subj.disruptionEffect:Targetness_	0.07	ρ_01 Subj.disr.Effect:Targetn_	−0.39
Conditional *R*^2^:	NA	τ_1 Subj.recoveryEffect:Targetness_	0.03	ρ_01 Subj.recoveryEffect:Targetn_	0.63

*All values are log-scaled. The bold values indicate that the effect was statistically significant.

### Stem Completion Task

**FIGURE 3:**
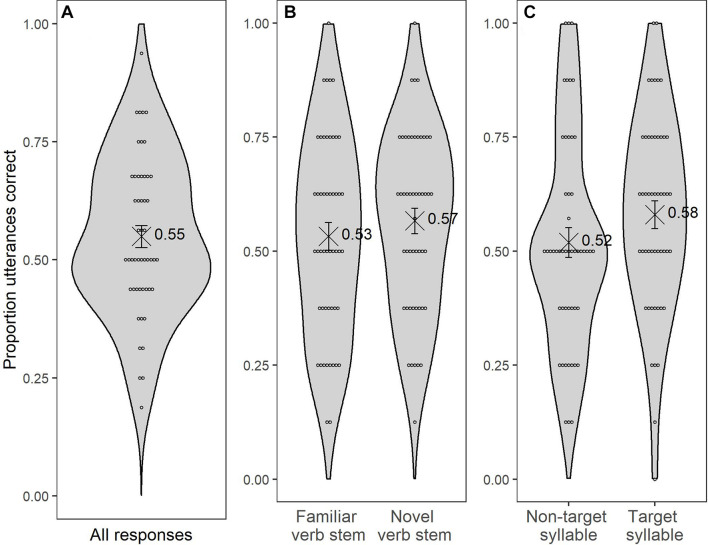
Violin plots that represent the distribution of **(A)** the overall mean accuracy scores on the SC task, **(B)** the mean accuracy scores by *Familiarity*, and **(C)** the mean accuracy scores by *Targetness*. Error bars indicate SEs. The dots represent the individual scores and the black cross indicates the mean with its numerical value.

We tested whether children’s accuracy scores exceeded chance level (intercept at grand average across predictor levels significantly different from 0/50% probability) and whether their performance was influenced by *Targetness* and/or *Familiarity*, and whether there was an interaction with *Age*. Overall, children chose the correct stem with an accuracy of 54.94%, with individual accuracy scores ranging from 18.75 to 93.75%. Figure 3A shows children’s individual accuracy scores along with the overall mean accuracy score for the SC task. Figures 3B,C show the scores according to *Familiarity* and *Targetness*, respectively. The complete output of the model is provided in Table 6. The corresponding estimates show that children scored significantly above chance level [intercept: log-odds = 0.22, *z* = +2.17; *p* = 0.03; 95% CI_prob_ (50.5, 60.3%)]. There were no significant differences between trials with familiar verb stems and trials with novel verb stems [*Familiarity*, log-odds = −0.15, *z* = −1.02; *p* = 0.30; 95% CI (−0.45, 0.14)] nor between target and non-target trials [*Targetness*, log-odds = 0.26, *z* = 1.43; *p* = 0.15; 95% CI (−0.11, 0.63)]. Therefore, we cannot conclude that children treat familiar items differently from novel ones or targets differently from non-targets. Moreover, there was no main effect of *Age nor* did *Age* significantly modulate the effects. No other interactions in the model yielded statistically significant effects (see Table 6). Finally, we used the method proposed by Lammertink et al. (2019, 2020) to explore whether children’s performance in the SRT task correlated with their performance in the SC task. The Pearson’s correlation coefficient was not statistically significantly different from zero (*r* = 0.11, *p* = 0.45).

**TABLE 6:** Summary of the accuracy model for the SC task (799 observations, *N* = 50).

Accuracy						
Predictors	Log-odds	CI (log-odds)	Odds ratios	CI (odds ratios)	*z*-Statistic	*p*-Value
(Intercept)	0.22	(0.02 to 0.42)	1.25	(1.02 to 1.52)	**2.17**	**0.030**
Targetness	0.26	(−0.11 to 0.63)	1.30	(0.90 to 1.88)	1.43	0.153
Age (centered)	0.11	(−0.07 to 0.29)	1.12	(0.93 to 1.34)	1.24	0.215
Familiarity	−0.15	(−0.45 to 0.14)	0.86	(0.64 to 1.15)	−1.02	0.306
Targetness* Age (centered)	0.15	(−0.17 to 0.48)	1.16	(0.84 to 1.62)	0.93	0.352
Familiarity* age (centered)	−0.13	(−0.38 to 0.13)	0.88	(0.68 to 1.14)	−0.96	0.335

**Random effects**						

σ^2^	3.29					
τ_0 Subj_	0.22					
τ_1 Subj.Targetness_	0.57					
ρ_01 Subj.Targetness__1_	−0.24					
ICC	0.10					
N _*Subj*_	50					
Observations: 799	Marginal *R*^2^/conditional *R*^2^: 0.015/0.112					

*All values are log-scaled. The bold values indicate that the effect was statistically significant.

## Discussion

In the present study, we examined children’s ability to learn NADs within an active word-monitoring SRT task set up as a web-based computer game (*whack-a-mole*) with the objective of measuring NAD sensitivity in novel natural language stimuli. In short, our findings suggest that children between 4 and 8 years of age were sensitive to the internal rule-based structure of the two presented NADs and showed learning in both the SRT task as measured *via* RTs as well as learning in the SC task as measured *via* response accuracy.

Our findings indicate that at the group level, children in our study were able to learn the internal rule structure of both the target as well as the non-target NAD stimuli. Successful learning in the SRT word-monitoring task was indicated by: (1) a decrease in RTs during the first two learning blocks (**training effect**), suggesting that over time the correct (word-monitoring) responses to the second element of the NAD were predicted by the first element of the NAD, and by (2) an increase in RTs during the disruption block (**disruption effect**), in which violated NADs were presented (i.e., the prediction of the second element of the NAD was unreliable and thus led to increased RTs). Hence, we replicated the results of Lammertink et al. (2019, 2020) in a web-based setting. As expected, children showed overall faster responses for the target trials than the non-target trials, suggesting that children have learned the NAD related to the target syllable better compared to the non-target one. This may be due to the explicit wording of the instructions (“If you hear *are*, press the white button. If you don’t hear *are*, press the red button”). Finally, our data did not show the expected **recovery effect** (i.e., RTs did not go back to baseline after the disruption block). The observation of this effect would have been a further indicator for NAD learning. This can be due to a possible lack of power caused by too much variance in the data. Alternatively, some possible reasons for the absence of this effect are that children may need more trials to “recover” from the disruption block or that they were tired and/or less attentive toward the end of the experiment, or that they were surprised in the disruption block, and therefore their recovery was weakened or hindered.

Children in the present study were also asked to complete a SC task. Success in this task required children to apply or transfer the internal rule structure of the NAD, which was learned during the SRT task, to actively access the missing second element of the NAD to the given first element and verb stem. Our results show that children’s performance at the group level differed significantly from chance level, independently of targetness or familiarity of the stimuli. Children chose the correct second element of the NAD (*are* or *ando*) significantly more often than would be expected if they were only guessing. While children’s responses indicated at the group level that learning was achieved, we found large differences at the individual level, which were unrelated to age. Our SC task was a modification of the 2AFC task in Lammertink et al. (2019). In their 2AFC task participants had to decide which of the two presented utterances was more familiar to the previously heard utterances, and they failed to do so. 2AFC tasks of this kind have a high working memory load and can lead to *response biases* (Fritzley and Lee, 2003), such that children tend to provide only one type of answer (Okanda and Itakura, 2010). Hence, the SC task as used in our study might be a more suitable task for showing explicit NAD sensitivity in children, despite the high variability in the data. Finally, we have no evidence that children’s SC accuracy scores could explain the variance in their SRT data because the correlation between the learning in the two tasks did not reach significance. The reason for this might be that the two tasks measure learning in different ways (implicit learning vs. accessing the knowledge more explicitly), therefore relating both might not address the same information. Also, it should be acknowledged that the lower number of trials in the SC task might distort potential effects, and therefore the SC results need to be interpreted with caution.

An additional goal of the present work was to assess whether children’s NAD learning is affected by age. We found no evidence that age modulated the training effect or the disruption effect in the age range tested in our study. However, we found a main effect of age, which indicated that younger children, as expected, generally responded slower in the SRT task than older children. In addition, there was a significant interaction between age and targetness (target and non-target). That is, the youngest children (4-year-olds) were slower in responding to target items than to non-target items, while the opposite was determined for the oldest children from the sample. Furthermore, the RT distribution of the youngest children was more spread out and thus more data points from these children were excluded than from older ones. However, it is likely that this would also be the case in laboratory studies. Therefore, conclusions on the influence of age on RTs need to be considered with special caution. Our results are in line with Lammertink et al. (2019, 2020), who also showed that older children can learn NADs in this behavioral setting. In contrast to our hypothesis, we did not observe any sign of decreased sensitivity to the NADs for older children compared to younger ones, as could have been expected from the suggested developmental shift caused by the maturation of the PFC (Skeide and Friederici, 2016). A possible explanation could be that the SRT task can be considered an active task, although the NADs are not explicitly mentioned in the instruction. It might also be that case that older children coped better with the attentional and motoric demands of the web-based setting and therefore compensated for a potential age effect. We can conclude from our data that, at the group level, children between 4 and 8 years of age can learn NADs if they are assigned an active task like the one in this study (monitoring a word). We believe that future studies should explore possible underlying cognitive processes by means of brain-structural or -functional indices and should continue to address implicit vs. explicit learning in older age ranges, for example, by adding instructions pointing to the internal structure of the stimuli.

To our knowledge, our study is the first testing children with an SRT task in a web-based setting. Here, we did not observe any substantial differences between our results from a web-based study and a similar laboratory-based experiment, as in Lammertink et al. (2019, 2020). Our study therefore demonstrates that RTs collected *via* the web with children aged 4–8 years is feasible and delivers reliable results. While running this study fully online had the advantage of allowing us to collect data during the global pandemic and in a faster manner compared to the laboratory-based sample collection, this procedure still poses particular methodological challenges. Firstly, there are several influencing factors that cannot be controlled in the same way as in the laboratory. For example, parents were required to prepare the keyboard, assure the appropriate surroundings for their children (quiet room without distractions, appropriate volume, etc.) and were asked not to help or assist in the completion of the tasks. We presume that these prerequisites were met, but we cannot verify this. Secondly, we encountered very long RTs, especially in younger children. The three youngest participants of our sample (aged 3;7 to 4;18) showed RTs longer than 7000 ms after stimulus offset in more than 12% of their responses. We believe that such long RTs do not reflect linguistic stimulus processing and we therefore applied different outlier criteria than typically used in a laboratory-based study (e.g., set an upper cut-off to 7000 ms and excluded values at 2.5 SD from the mean). One possibility to avoid these challenges of web-based experiments in further studies would be conducting moderated studies including a debriefing with the experimenter (at the cost of privacy data protection) or to include a time-out for the single trials. However, we found no evidence that young children in our sample had a different learning behavior than the older children and thus we assume that their large range of RTs is most likely due to their shorter attention span compared to older children and not indicative of deficits in NAD learning. Furthermore, we had to exclude a substantial number of datasets from our analyses, because children did not follow the task instruction (and pressed randomly in the word-monitoring SRT task) – a behavior that can be better monitored in a laboratory-based experiment. In future studies, those children could receive an additional practice block or instructions, or the experiment could be stopped, for instance, after the first learning block. Secondly, accuracy of RTs in a web-based setting may sometimes be considered unprecise and unreliable (Germine et al., 2012; Reimers and Stewart, 2015). Although we cannot fully control the RT precision of the *LabVanced* software, we did not notice any missing values or larger gaps in the distribution due to the software. In addition, *LabVanced* developers attest that the collection and measurement of RTs are highly reliable. Importantly, the findings from our study rely on relative RTs and not absolute RTs, which makes the measurement more reliable. We therefore encourage further studies exploring small effect sizes with RTs to consider within-subject designs. Also, to confirm the reliability of the observed results, it would be beneficial to repeat the study in a laboratory setting with a similar age sample. In this context, the 4-year-old children are of special interest, because their RT data showed the largest range. In a laboratory setting, it would be possible to evaluate some of the reasons for this behavior regarding distractibility or difficulty in focusing over a longer period of time.

In conclusion, our study provides novel evidence on the learning of NADs in children aged 4–8 years in a web-based game-like task. Despite the variability between and within participants, our data suggest that the children are capable of learning NADs in an SRT task. In addition, the present work contributes evidence to web-based research demonstrating the feasibility of testing children online. Taking the discussed advantages and challenges into consideration, we believe that the use of online studies is a promising alternative or supplement to traditional laboratory-based studies.

## Data Availability Statement

The datasets presented in this study can be found in online repositories. The names of the repository/repositories and accession number(s) can be found below: https://osf.io/3u62p/.

## Ethics Statement

The studies involving human participants were reviewed and approved by the Ethics Committee of the University of Potsdam, Faculty of Humanities (EA 43/2018). Written informed consent to participate in this study was provided by the participants’ legal guardian/next of kin.

## Author Contributions

MM and AH were responsible for implementing the experimental paradigm, collecting and analyzing the data, and writing the manuscript. MM, AH, and JV were involved in data collection and data analysis. AF, CM, BH, and IW conceptualized and designed the study, discussed the implementation, and contributed to writing and revising the manuscript. All authors contributed to the article and approved the submitted version.

## Conflict of Interest

The authors declare that the research was conducted in the absence of any commercial or financial relationships that could be construed as a potential conflict of interest.

## Publisher’s Note

All claims expressed in this article are solely those of the authors and do not necessarily represent those of their affiliated organizations, or those of the publisher, the editors and the reviewers. Any product that may be evaluated in this article, or claim that may be made by its manufacturer, is not guaranteed or endorsed by the publisher.

## Footnotes

1 For reasons of privacy data protection, parents were asked to provide the year and month of their child’s birth before starting the experiment. In the analysis a date specification was compiled by setting the numeric of the birthday date to the 15th of each month. Thus, it is possible that children’s age deviates from the actual age by a maximum of 15 days. In addition, parents were asked to create a code that would serve as the ID for the data analysis. This ID was only linked to the raw data and could not be traced back to the actual participant.

2
www.kinderschaffenwissen.de

3 The *LabVanced*’s server is located in Germany, does not store any personal data, and follows the EU data privacy regulations (GDPR). More information can be found at the website https://www.labvanced.com/docs/geninfo/security/.

4 All operating systems were allowed. All Internet browsers were allowed, except Safari.

5
https://osf.io/bt8ug/
